# Role of TRPM2 in H_2_O_2_-Induced Cell Apoptosis in Endothelial Cells

**DOI:** 10.1371/journal.pone.0043186

**Published:** 2012-08-20

**Authors:** Lei Sun, Ho-Yan Yau, Wei-Yan Wong, Ronald A. Li, Yu Huang, Xiaoqiang Yao

**Affiliations:** 1 Li Ka Shing Institute of Health Sciences and School of Biomedical Sciences, The Chinese University of Hong Kong, Hong Kong, China; 2 Stem Cell and Regenerative Medicine Consortium, The University of Hong Kong, Hong Kong, China; Catholic University Medical School, Italy

## Abstract

Melastatin-like transient receptor potential channel 2 (TRPM2) is an oxidant-sensitive and cationic non-selective channel that is expressed in mammalian vascular endothelium. Here we investigated the functional role of TRPM2 channels in hydrogen peroxide (H_2_O_2_)-induced cytosolic Ca^2+^ ([Ca^2+^]_i_) elavation, whole-cell current increase, and apoptotic cell death in murine heart microvessel endothelial cell line H5V. A TRPM2 blocking antibody (TM2E3), which targets the E3 region near the ion permeation pore of TRPM2, was developed. Treatment of H5V cells with TM2E3 reduced the [Ca^2+^]_i_ rise and whole-cell current change in response to H_2_O_2_. Suppressing TRPM2 expression using TRPM2-specific short hairpin RNA (shRNA) had similar inhibitory effect. H_2_O_2_-induced apoptotic cell death in H5V cells was examined using MTT assay, DNA ladder formation analysis, and DAPI-based nuclear DNA condensation assay. Based on these assays, TM2E3 and TRPM2-specific shRNA both showed protective effect against H_2_O_2_-induced apoptotic cell death. TM2E3 and TRPM2-specific shRNA also protect the cells from tumor necrosis factor (TNF)-α-induced cell death in MTT assay. In contrast, overexpression of TRPM2 in H5V cells resulted in an increased response in [Ca^2+^]_i_ and whole-cell currents to H_2_O_2_. TRPM2 overexpression also aggravated the H_2_O_2_-induced apoptotic cell death. Downstream pathways following TRPM2 activation was examined. Results showed that TRPM2 activity stimulated caspase-8, caspase-9 and caspase-3. These findings strongly suggest that TRPM2 channel mediates cellular Ca^2+^ overload in response to H_2_O_2_ and contribute to oxidant-induced apoptotic cell death in vascular endothelial cells. Down-regulating endogenous TRPM2 could be a means to protect the vascular endothelial cells from apoptotic cell death.

## Introduction

Reactive oxygen species (ROS) are key factors in pathophysiology of vascular endothelial cells. Excessive production of ROS damages the structure and function of endothelial cells, leading to endothelial dysfunction [1], which may contribute to pathogenesis of hypertension, diabetes, inflammation and atherosclerosis [1], [2]. Evidence shows that ROS-induced endothelial dysfunction is often preceded by an alteration of endothelial [Ca^2+^]_i_ [3], which serves as an important second messenger to trigger apoptosis and cell death.

TRPM2 is a Ca^2+^-permeable nonselective cation channel. Its main endogenous gating molecule is adenosine 5′-diphosphoribose (ADP-ribose) [4], [5], [6]. Binding of ADP-ribose to TRPM2 opens the channel, allowing Na^+^ and Ca^2+^ to enter the cells. ADP-ribose activation of TRPM2 is potentiated by [Ca^2+^]_i_, nicotinic acid adenine dinucleotide phosphate and H_2_O_2_, which is a major ROS [6], [7], [8]. In addition to its potentiation effect, H_2_O_2_ may directly stimulate TRPM2 activity [9], [10]. It has been shown that H_2_O_2_-induced Ca^2+^ influx through TRPM2 contributes to ROS-induced cell death in several cell types including neuons, hematopoietic cells and TRPM2-overexpressing HEK293 cells [7], [11], [12], [13]. TRPM2-S is an TRPM2 isoform.TRPM2-S exerts dominant-negative effect on TRPM2 function, serving to inhibit H_2_O_2_-induced [Ca^2+^]_i_ rises and its associated cell death in TRPM2-expressing cells [12]. In cultured rat neurons, both TRPM2-S and TRPM2-specific siRNA were found to reduce H_2_O_2_-induced [Ca^2+^]_i_ rises and cell death [11]. Besides TRPM2, ROS could activate other Ca^2+^ influx channels and stimulate intracellular store Ca^2+^ release, contributing to Ca^2+^ overload and cell death [14], [15], [16].

TRPM2 is abundantly expressed in vascular endothelial cells [17], [18]. However, to date, there is only one report studying the role of TRPM2 in vascular endothelial cells [18]. In that study, Hecquet et al. demonstrated that ROS-induced TRPM2 activation may contribute to an increased vascular permeability [18]. However, some important questions remained unsolved, including: 1) whether TRPM2 activity plays a role in endothelial cell death, and 2) whether inhibiting TRPM2 could protect endothelial cells from ROS-induced cell death. In the present study, we address these questions using a heart microvessel endothelial cell line H5V [19]. Our results show that TRPM2 is a key molecule involved in H_2_O_2_-induced endothelial cell death and that inhibiting TRPM2 is an effective means to protect the endothelial cells from H_2_O_2_-induced cell death.

## Results

### Involvement of TRPM2 Channels in H_2_O_2_-induced Ca^2+^ Influx in H5V Cells

**Figure 1:**
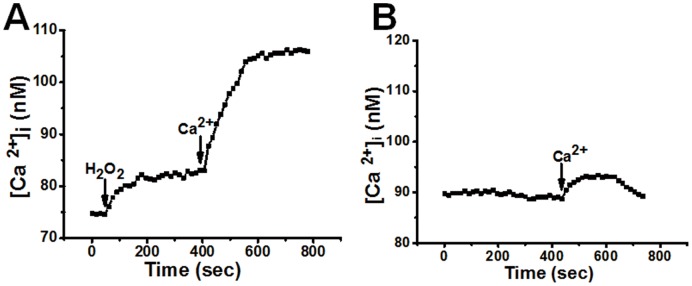
H_2_O_2_-induced Ca^2+^ influx in H5V cells A. Representative trace showing the [Ca^2+^]_i_ responses to H_2_O_2_ and extracellular Ca^2+^ add-back in H5V cells. Cells bathed in 0Ca^2+^-PSS were first challenged by 3 mM H_2_O_2_ followed by 2 mM extracellular Ca^2+^. The Ca^2+^ add-back induced an additional [Ca^2+^]_i_ rise, presumably due to Ca^2+^ influx (n = 4). **B.** Representative trace showing the [Ca^2+^]_i_ response to extracellular Ca^2+^ add-back in the absence of H_2_O_2_. The cells were bathed in 0Ca^2+^-PSS at the beginning, followed by 2 mM extracellular Ca^2+^ (n = 3).

H5V cells were bathed in a Ca^2+^-free solution (0Ca^2+^-PSS). Application of H_2_O_2_ (3 mM) initiated a [Ca^2+^]_i_ rise, presumably due to H_2_O_2_-induced Ca^2+^ release from the intracellular Ca^2+^ stores (Fig. 1A). Ca^2+^ was then added to the extracellular bath, causing another [Ca^2+^]_i_ rise (Fig. 1A). This second [Ca^2+^]_i_ rise was mostly due to H_2_O_2_-induced Ca^2+^ influx. In the absence of H_2_O_2_, the Ca^2+^ add-back to the bath only had very small effect on [Ca^2+^]_i_ level (Fig. 1B).

**Figure 2:**
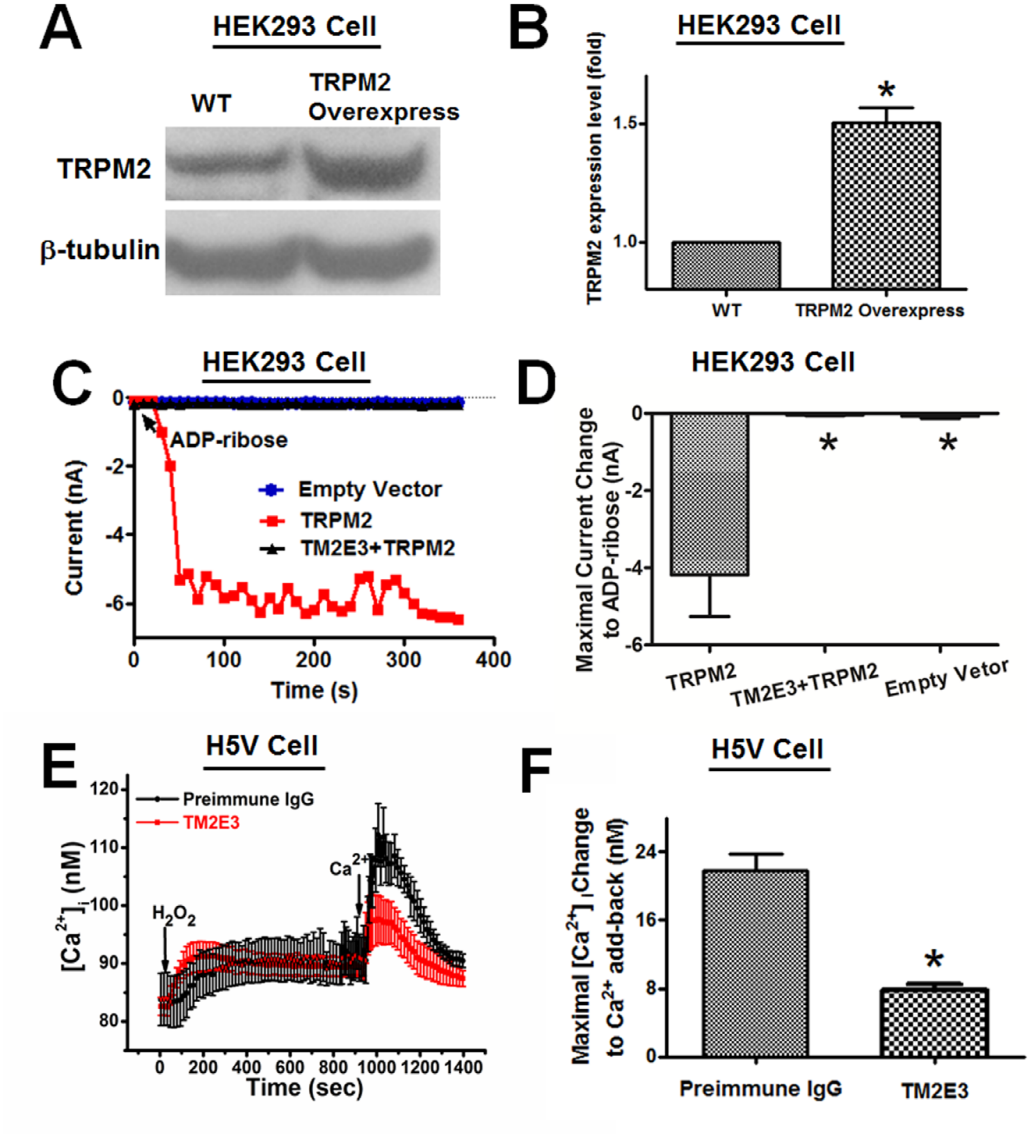
Effect of TM2E3 on H_2_O_2_-induced Ca^2+^ influx in H5V cells and on ADP-ribose-activated whole-cell currents in HEK293 cells. A and B. Representative immunoblots (A) and data summary (B) probed with TM2E3 and anti-β-tubulin. Whole cell lysates were taken from wild-type HEK293 cells (WT) and TRPM2-overexpresssing HEK293 cells (TRPM2 Overexpress). In B, the expression level of TRPM2 in WT was normalized to 1. (n = 4), *P<0.05 compared with WT. **C and D.** Representative traces (C) and data summary (D) showing the effect of TM2E3 on the whole-cell currents activated ADP-ribose. Recordings were made in HEK293 cells that were transfected with TRPM2 or empty plasmid. ADP-ribose (300 µM) was included in pipette solution. Cells were hold at −100 mV. (n = 4−6), *P<0.05 compared with TRPM2 group. **E and F.** Representative traces (E) and data summary (F) showing the effect of TM2E3 on H_2_O_2_-induced Ca^2+^ entry in H5V cells. Cells were pretreated with TM2E3 (10 µg/ml) or Pre-immune IgG (10 µg/ml) for 1 hr. The cells were first bathed in 0Ca^2+^-PSS, followed by 3 mM H_2_O_2_, and then 2 mM Ca^2+^ add-back. Maximal [Ca^2+^]_i_ changes in response to 2 mM Ca^2+^ add-back were shown in F. n = 5, *P<0.05 compared with Pre-immune IgG group.

**Figure 3:**
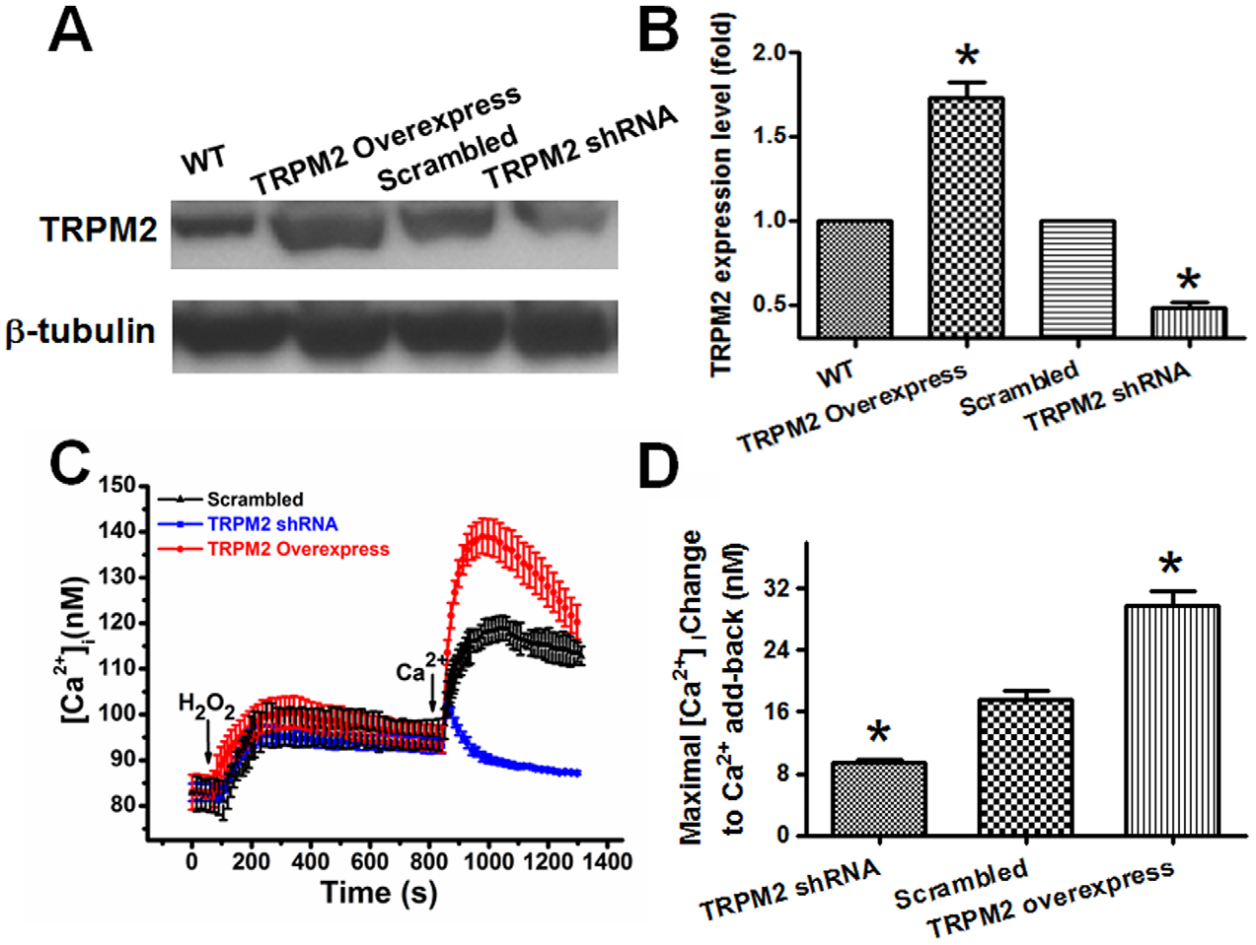
Effect of TRPM2-specific shRNA on H_2_O_2_-induced Ca^2+^ influx in H5V cells. A and B. Representative immunoblots (A) and data summary (B) showing the effectiveness of TRPM2-specific shRNA in suppressing TRPM2 expression in H5V cells. H5V cells were transfected with or without TRPM2, scrambled-shRNA, or TRPM2-specific shRNA (n = 3 experiments), *P<0.05 compared with WT or scrambled-shRNA. **C and D.** Representative traces (C) and data summary (D) showing the effect of TRPM2-specific shRNA on H_2_O_2_-induced Ca^2+^ entry in H5V cells. The cells were first bathed in 0Ca^2+^-PSS, followed by 3 mM H_2_O_2_, and then 2 mM Ca^2+^ add-back. Maximal [Ca^2+^]_i_ changes in response to 2 mM Ca^2+^ add-back were shown in D. n = 5, *P<0.05 compared with scrambled-shRNA.

A blocking antibody targeted against E3-region near the permeation pore of TRPM2 channels was developed using the strategy reported elsewhere [20]. The specificity of TM2E3 was verified by immunoblots (Figs. 2A and B) and patch clamp (Figs. 2C and D). TM2E3 could recognize the specific TRPM2 band in TRPM2-overexpressing HEK293 cells as demonstrated in immunoblots (Figs. 2A and B). In the patch clamp study, TM2E3 (10 µg/ml, 1 hr pretreatment) could effectively block TRPM2-mediated whole-cell currents in response to ADP-ribose in TRPM2-overexpressing HEK293 cells (Figs. 2C and D). In H5V cells, TM2E3 inhibited H_2_O_2_-induced Ca^2+^ influx, which was the second [Ca^2+^]_i_ rise in response to Ca^2+^ add-back (Figs. 2E and F). A TRPM2-specific shRNA was designed. In immunoblots, the TRPM2-specific shRNA could effectively reduce the expression of TRPM2 proteins in H5V cells (Figs. 3A and B). In [Ca^2+^]_i_ study, the TRPM2-specific shRNA markedly reduced the H_2_O_2_-induced Ca^2+^ influx whereas TRPM2 overexpression significantly augmented the Ca^2+^ influx to H_2_O_2_ in H5V cells (Figs. 3C and D). These data suggest a key functional role of TRPM2 channels in mediating H_2_O_2_-induced Ca^2+^ influx in H5V cells.

### Involvement of TRPM2 Channels in H_2_O_2_-elicited Whole-cell Current in H5V Cells

**Figure 4:**
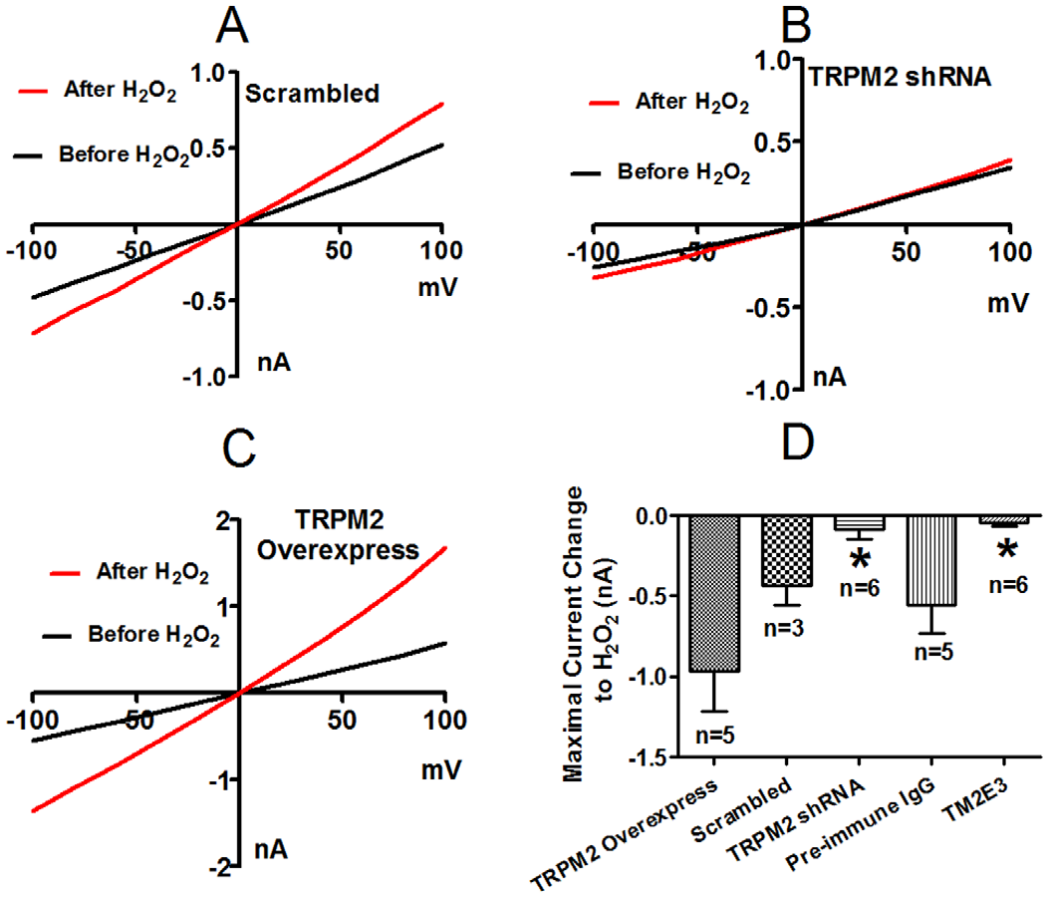
Effect of TRPM2-specific shRNA and TM2E3 on H_2_O_2_-elicited whole-cell current change in H5V cells. Representative whole-cell current-voltage (I-V) relationships before and after H_2_O_2_ (3 mM) treatment in H5V cells transfected with scrambled-shRNA (**A**), TRPM2-specific shRNA (**B**) and TRPM2 (**C**). Whole-cell currents were recorded using linear ramp protocol from −100 mV to +100 mV with 100 ms duration. (**D**) Maximal changes in inward currents in response to 3 mM H_2_O_2_ at −100 mV. The changes were obtained by subtracting the current before H_2_O_2_ treatment from that after the treatment. n = 3−6, *P<0.05, compared with scrambled-shRNA or pre-immune IgG.

Whole-cell currents were measured by patch clamp. Extracellular application of H_2_O_2_ (3 mM) caused an increase in whole-cell currents in H5V cells (Fig. 4A). The H_2_O_2_-elicited whole-cell currents displayed a linear current-voltage relationship, which is typical of TRPM2 [4], [7], [18]. The H_2_O_2_-elicited whole-cell currents were markedly attenuated in H5V cells that were pretreated with TM2E3 (10 µg/ml, 1 hr) or transfected with TRPM2-specific shRNA (Figs. 4B and D). In contrast, the currents increased in cells that were overexpressed with TRPM2 (Figs. 4C and D). These data indicate that TRPM2 accounts for a large part of H_2_O_2_-elicited whole-cell currents in H5V cells.

### Role of TRPM2 Channels in H_2_O_2_-induced Apoptotic Cell Death in H5Vcells

**Figure 5:**
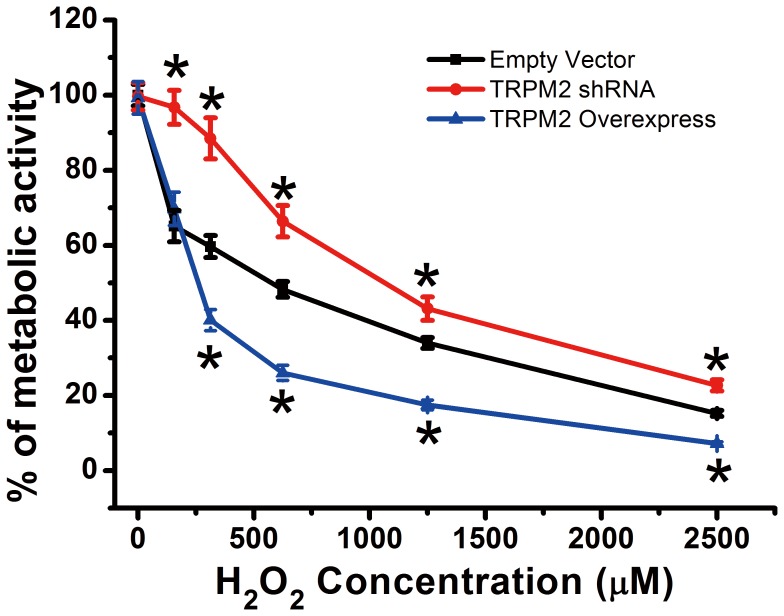
TRPM2-mediated loss of cell metabolic activity in response to H_2_O_2_ in H5V cells. H5V cells that were stably transfected with empty vector, TRPM2-specific shRNA or TRPM2 were treated with indicated concentrations of H_2_O_2_ (1, 156, 312, 625, 1250, 2500 µM, respectively) for 1 hr. Cell metabolic activity was assessed by MTT assay. The data were expressed as the percent of metabolic activity in the absence of H_2_O_2_. n = 6 per group, *P<0.05 compared with Empty Vector.

Loss of metabolic activity in H5V cells was measured by MTT assay. H_2_O_2_ treatment for 1 hr caused a reduction in metabolic activity in a concentration-dependent manner. H_2_O_2_ concentration ranged from 1, 156, 312, 625, 1250, to 2500 µM (Fig. 5). Overexpression of TRPM2 augmented the H_2_O_2_-induced loss of metabolic activity, whereas TRPM2-specific shRNA partially reversed the damaging effect of H_2_O_2_ (Fig. 5).

**Figure 6:**
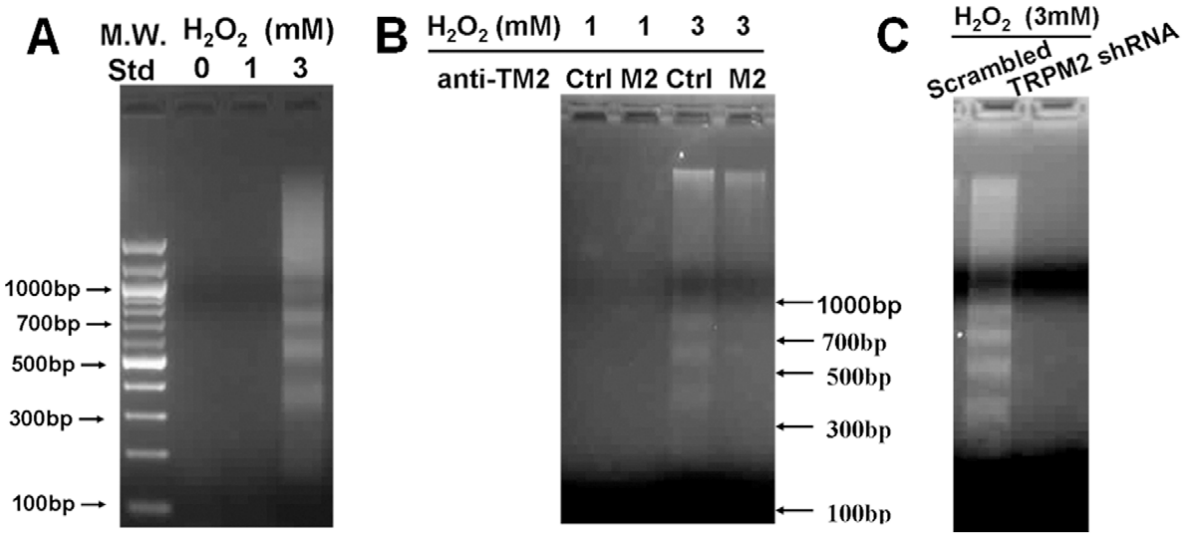
Role of TRPM2 in H_2_O_2_-induced H5V cell apoptosis as assessed by DNA fragmentation. **A.** Dose-dependent effect of H_2_O_2_ on DNA fragmentation. H5V cells were treated without indicated concentration of H_2_O_2_ for 24 hr. DNA was extracted and analyzed by agarose gel electrophoresis. Molecular weight standard (M.W. Std) was shown. **B.** Inhibitory effect of TM2E3 on H_2_O_2_-induced DNA fragmentation. Cells were pretreated with pre-immune IgG (labeled as Ctrl) or TM2E3 (labeled as M2) for 2 hr before treatment of indicated concentration of H_2_O_2_ for 24 hr. **C.** Effect of TRPM2-specific shRNA on H_2_O_2_-induced DNA fragmentation. Cells were transfected with scrambled-shRNA or TRPM2-specific shRNA. No DNA fragmentation was observed in cells transfected with TRPM2-specific shRNA. n = 3 per group.

H_2_O_2_-induced apoptotic cell death of H5V cells was determined by DNA fragmentation. H_2_O_2_ at 3 mM was able to induce DNA fragmentation, indicated by appearance of a “ladder” pattern at ∼180 bp interval (Fig. 6A). No such ladder pattern was observed in the absence of H_2_O_2_ treatment or when H_2_O_2_ concentration was lower (1 mM) (Fig. 6A). Prominently, treatment of cells with TM2E3 or TRPM2-specific shRNA reduced the DNA fragmentation in response to 3 mM H_2_O_2_ (Figs. 6B and C).

**Figure 7:**
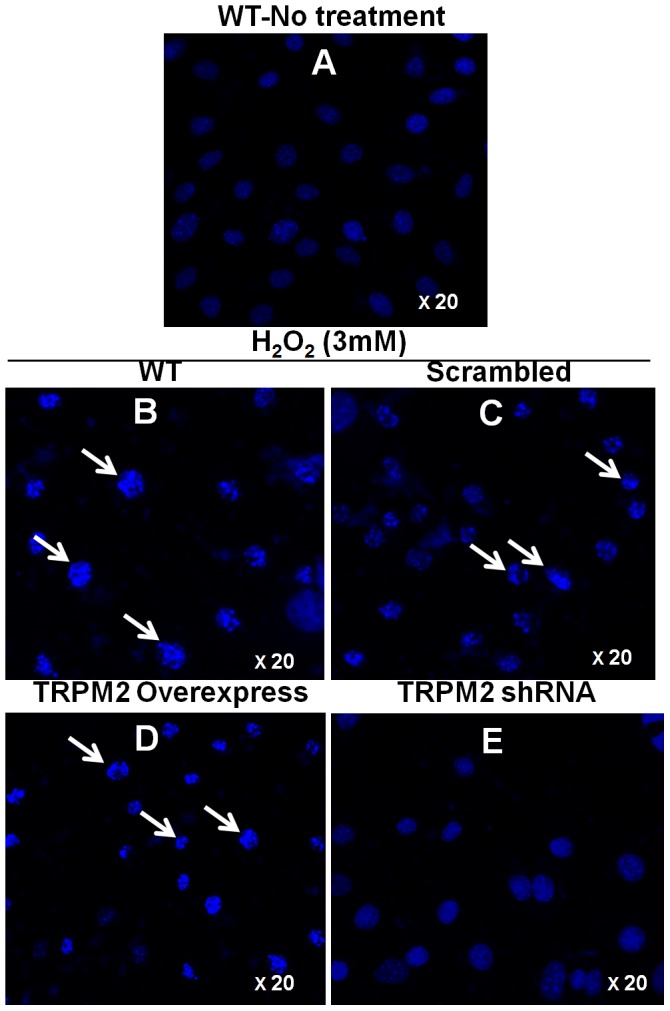
Role of TRPM2 in H_2_O_2_-induced H5V cell apoptosis as assessed by DAPI staining. Shown were nuclear morphology as detected by DAPI (1 µg/ml) staining (blue) under fluorescence microscope. **A.** Wild-type H5V cells without H_2_O_2_ treatment. **B-E.** After 24 hr treatment with 3 mM H_2_O_2_ for wild-type H5V cells (**B**), scrambled-shRNA-transfected H5V cells (**C**), TRPM2-overexpressing H5V cells (**D**) and TRPM2-specific shRNA-transfected H5V cells (**E**). White arrows indicate the nuclei with condensed chromatin and fragmented nuclear bodies. n = 6 per group. UV wavelength, 340 nm; Exposure time, 200 ms.

The role of TRPM2 channels in H_2_O_2_-induced apoptotic cell death was morphologically confirmed by a fluorescent DNA-binding agent DAPI staining. Representative nuclear morphology of H5V cells were shown in Fig. 7A. Control cells without H_2_O_2_ treatment displayed clear-edged, uniformly stained blue nuclei. After H_2_O_2_ treatment for 24 hr, nuclear condensation and nuclear fragmentation, both of which are characteristic of apoptotic cells, were observed in non-transfected H5V cells (Fig. 7B) and scrambled-shRNA transfected H5V cells (Fig. 7C). The H_2_O_2_-induced nuclear condensation appeared to be aggravated in TRPM2-overexpressing cells (Fig. 7D). In contrast, H_2_O_2_-induced nuclear condensation was remarkably reduced in cells stably expressing TRPM2-specific shRNA (Fig. 7E).

### Involvement of Caspases

**Figure 8:**
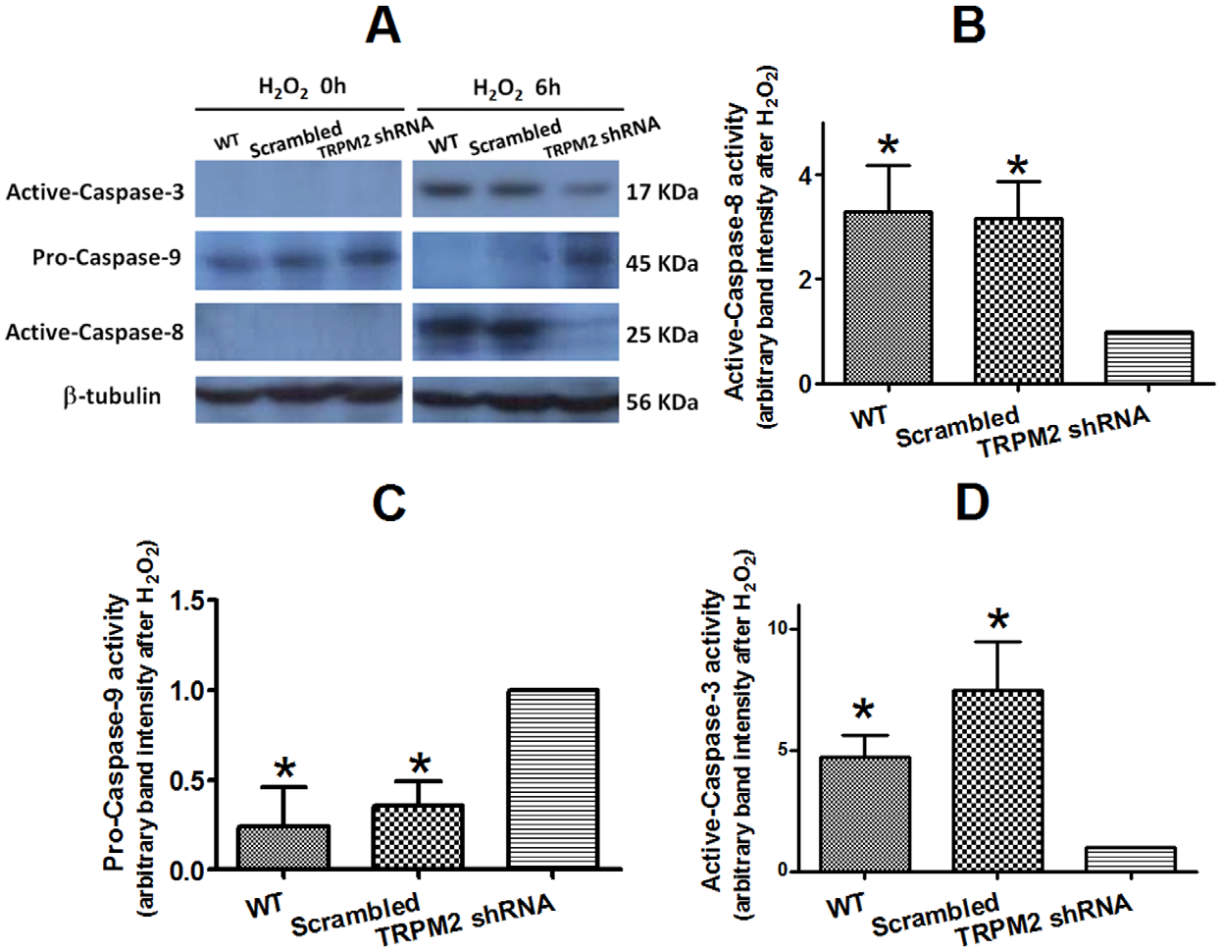
Effect of TRPM2-specific shRNA on H_2_O_2_-induced caspase activation in H5V cells. A. Representative immunoblots showing H_2_O_2_-induced activation of caspase-3, caspase-8 and caspase-9. H5V cells were transfected with or without scrambled-shRNA or TRPM2-specific shRNA. Left panels, before H_2_O_2_; right panels, 6 hr after 3 mM H_2_O_2_. Caspase-3 and caspase-8 activation was indicated by elevation of cleaved caspase-3 (active form, 17 kDa) and caspase-8 (active form, 25 kDa) after H_2_O_2_, while caspase-9 activation was illustrated by reduction of procaspase-9 (45 kDa). (**B-D**) Data summary showing the inhibitory effect of TRPM2-specific shRNA on H_2_O_2_-induced activation of caspase-8 (B), caspase-9 (**C**) and caspase-3 (**D**). The caspase expressional level in cells expressing TRPM2-specific shRNA was normalized to 1. n = 4 per group, *P<0.05 compared with TRPM2 shRNA.

Effect of H_2_O_2_ on the activity of caspase-3, caspase-8 and caspase-9 was investigated. Treatment of H5V cells with H_2_O_2_ (3 mM, 6 hrs) activated caspase-3 and caspase-8 based on the elevation of caspase-3 and caspase-8 level in immunoblots (Fig. 8). The H_2_O_2_ treatment caused a reduction of procaspase-9 level, suggesting that H_2_O_2_ also activated caspase-9 (Fig. 8). In cells that were transfected with TRPM2-specific shRNA, the effect of H_2_O_2_ on caspase level was much reduced (Fig. 8).

### Involvement of TRPM2 in TNF-α-induced Cell Death in H5V Cells

**Figure 9:**
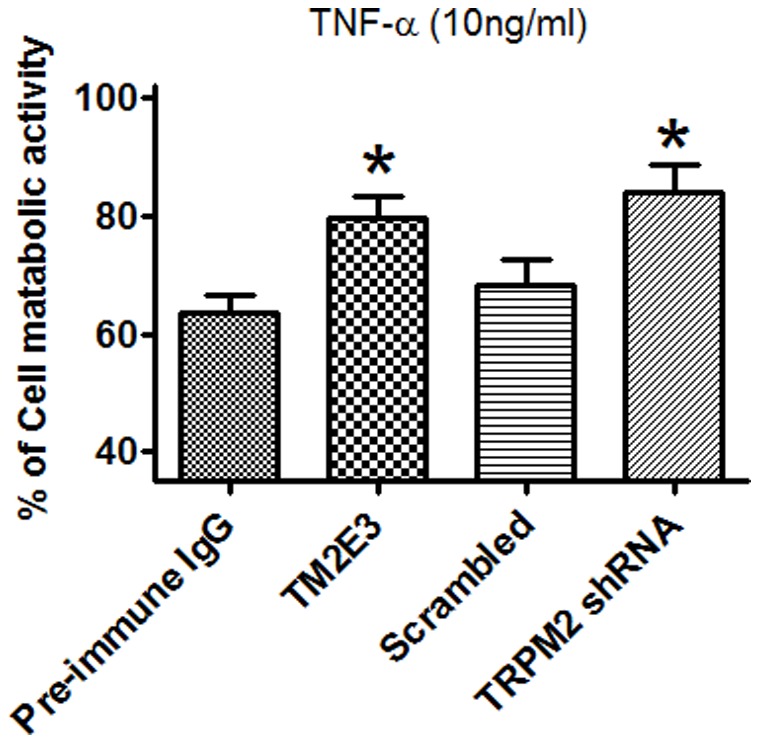
Role of TRPM2 in TNF-α-induced cell death in H5V cells. H5V cells that were stably transfected with scrambled-shRNA or TRPM2-specific shRNA. These cells were exposed to 10 ng/ml of TNF-α for 36 hrs. In another series, wild-type H5V cells were pretreated with TM2E3 (10 µg/ml) or Pre-immune IgG (10 µg/ml) for 2 hrs before TNF-α challenge. The cell metabolic activity was assessed by MTT assay. The data were expressed as the percent of metabolic activity in the absence of TNF-α. n = 6 per group, *P<0.05 compared with scrambled-shRNA (for TRPM2-shRNA) or Pre-immune IgG (for TM2E3).

TNF-α is an inflammatory cytokine that can trigger H_2_O_2_ formation in endothelial cells [21], [22]. In MTT assay, treatment of H5V cells with TNF-α (10 ng/ml, 36 hrs) caused a loss of cell metabolic activity (Fig. 9). The TNF-α-induced cell death was attenuated in cells that were treated with TM2E3 or TRPM2-specific shRNA (Fig. 9).

## Discussion

ROS-induced endothelial cell damage leads to several diseases including hypertension, diabetes, inflammation and atherosclerosis [1], [2]. In the present study, we found that H_2_O_2_ could elicit [Ca^2+^]_i_ rises, induce whole-cell currents and trigger apoptotic cell death. Blocking or suppressing TRPM2 markedly reduced the H_2_O_2_-induced [Ca^2+^]_i_ rises and whole-cell currents. More importantly, blocking or suppressing TRPM2 inhibited the H_2_O_2_-induced cell death in H5V endothelial cells. We also explored the involvement of TRPM2 in TNF-α-induced cell death. TNF-α is an inflammatory cytokine that can potently induce intracellular ROS formation in endothelial cells, leading to apoptotic cell death and atherosclerotic development [21], [22]. Our results also demonstrated the role of TRPM2 in TNF-α-induced endothelial cell death. Taken together, these data for the first time demonstrated a key role of TRPM2 in H_2_O_2_-induced cell death in vascular endothelial cells and suggest an exciting possibility of targeting TRPM2 as an option for protecting of endothelial cells from ROS-induced cell damage.

With the use of TM2E3 and TRPM2-specific shRNA, we demonstrated that TRPM2 mediates the [Ca^2+^]_i_ responses to H_2_O_2_ in H5V cells. Once Ca^2+^ influx through TRPM2 is activated, resultant [Ca^2+^]_i_ rises are expected to trigger oxidative stress-induced cell death through a number of well-characterized pathways including caspases cleavage and poly-ADP-ribose Polymerase (PARP) inactivation [23]. Three different approaches, including MTT assay, DNA fragmentation and DAPI staining for nuclear DNA condensation, were used to monitor the H_2_O_2_-induced apoptotic cell death. MTT assay measures the loss of metabolic activity of cells and is an early indicator for cell death [24], [25]. DNA fragment is a hallmark of apoptosis with cleavage of chromatin DNA into internucleosomal fragments of 180 bp and multiples thereof [26]. DAPI staining for nuclear DNA condensation is another standard technique for apoptotic cell death [27]. In apoptotic cells, the integrity of the cell membrane is compromised. Therefore, more DAPI enters the cells, resulting in a stronger staining color of nuclear DNA. In the present study, we found that H_2_O_2_ and TNF-α caused a loss of metabolic activity in H5V cells as determined by MTT assay. H_2_O_2_ also caused DNA fragmentation based on DNA ladder assay, and induced nuclear DNA condensation in DAPI staining experiments. Importantly, these apoptotic responses to H_2_O_2_ and TNF-α were at least partially rescued by silencing TRPM2 expression with TRPM2-specific shRNA or blocking TRPM2 with specific antibody TM2E3. Effectiveness of TRPM2-specific shRNA was remarkable. After TRPM2-specific shRNA treatment, there was no apparent DNA ladder formation and nuclear DNA condensation. Together, these data established a firm linkage between TRPM2 and H_2_O_2_-induced apoptotic cell death in H5V cells.

We also investigated the downstream events following TRPM2 activation in the H_2_O_2_-induced endothelial cell apoptotic process. Involvement of caspase-3, caspase 8 and caspase-9 was studied. Caspsase-8 plays a central role in the extrinsic cell death pathways involving transmembrane receptor-mediated interactions, whereas caspase-9 is an important component in the intrinsic cell death pathway [28]. Caspase-3 is downstream of caspase-8 and caspase-9, and is activated by either initiator caspase (caspase-8 or caspase-9) [28]. Caspase 3 then acts as the executioner caspases, leading to the cell apoptosis [28]. Our data showed that H_2_O_2_ treatment activated all three caspases (caspase-8, caspase-9 and caspase-3). Importantly, suppressing TRPM2 by TRPM2-specific shRNA could reduce the activation of all three caspases. These results suggest H_2_O_2_, through its action on TRPM2, activates both intrinsic and extrinsic apoptosis pathways, leading to activation of caspases-3 and subsequent apoptosis. It is conceivable that TRPM2-mediated Ca^2+^ entry plays a key role in the process.

However, note that TM2E3 and TRPM2-specific shRNA could only partially inhibit the H_2_O_2_-induced [Ca^2+^]_i_ rises. Further, these two agents only partially rescued the endothelial cells from H_2_O_2_-induced loss of metabolic activity in MTT assay. We reason that there may exist some TRPM2-independent components that were also sensitive to H_2_O_2_. Other potential ROS-sensitive Ca^2+^-permeable channels have been reported. These include TRPC1 [29], TRPC3 and/or TRPC4 [30], TRPC5 [31], TRPC6 [32] and TRPM7 [15]. Further studies are needed to clarify which pathways and/or channels are also involved in H_2_O_2_-induced [Ca^2+^]_i_ rise and cell death in vascular endothelial cells.

We have used high concentration of H_2_O_2_ (3 mM) to induce apoptotic cell death in H5V microvessel endothelial cells and examined the role of TRPM2 in the process. It has been reported that high concentration of H_2_O_2_ was necessary in order to shorten the time for the terminal pathological events to occur [33] and to study the electrophysiological and [Ca^2+^]_i_ change as the acute consequences of the oxidative stress [12], [33]. Due to similar reason, many previous studies also used high concentration of H_2_O_2_ to study the acute effect of oxidative stress on endothelial cells [34], [35], [36]. Another contributing factor for using high concentration of H_2_O_2_ was that, in most of our studies, H_2_O_2_ treatment was carried out in culture media which contained serum. Serum, in particular albumin, has a strong quenching effect that drastically reduces levels of biologically active H_2_O_2_ [37]. We also tested the effect of relatively low concentration of H_2_O_2_ (100 µM-1 mM). However, at these concentration, H_2_O_2_ failed to induce [Ca^2+^]_i_ rises and cell apoptosis, suggesting that H5V cells were highly resistant to H_2_O_2_. In experiments involving DNA fragmentation and DAPI staining, the cells were incubated with H_2_O_2_ for relatively long period of time (24 hr) before DNA fragmentation and DAPI staining assays. As mentioned, H_2_O_2_ is rapidly degraded in the presence of serum. The long period of time before the assays was for development of the phenotypes (DNA fragment and nuclear condensation) rather than a requirement for long period of H_2_O_2_ treatment.

In conclusion, our study provides strong evidence for functional role of TRPM2 in H_2_O_2_-induced apoptotic cell death in vascular endothelial cells. In light of the role of oxidative stress in the initiation and/or progression of vascular diseases, we believe that TRPM2 channels may represent a potential therapeutic target for the treatment of ROS-mediated endothelial dysfunction and vascular diseases.

## Materials and Methods

### Cell Culture

A mouse heart endothelioma (H5V) cell line, a generous gift from Professor P. Huber [38], was derived from murine embryonic heart endothelium. H5V cells are a commonly used model for endothelial cell research [39], [40]. H5V cells and HEK293 cells were cultured in Dulbecco’s modified Eagle medium (DMEM) (Gibco, invitrogen, USA) supplemented with 10% Fetal Bovine Serum (Gibco, invitrogen, USA) and a combination of penicillin-streptomycin (Gibco, Invitrogen, USA) at 37°C in a 5% CO_2_ atmosphere.

### [Ca^2+^]_i_ measurement

H5V cells (non-transfected or those stably transfected with TRPM2, scrambled-shRNA or TRPM2-specific shRNA) were prepared and loaded with fluorescence dye Fura-2/AM (Molecular Probes, Inc., NJ) for observing their Ca^2+^ responses to H_2_O_2_ (VWR International Ltd., England). Briefly, cells were seeded on circular glass plates at 37°C overnight supplemented with culture medium. As for fluorescence dye loading, cells were incubated for an hour in dark at room temperature with 10 µM of Fura-2/AM and 0.02% Pluronic acid F-127 in normal physiological saline solution (N-PSS), which contained in mM: 1 CaCl_2_, 140 NaCl, 5 KCl, 1 MgCl_2_, 10 glucose, and 5 Hepes at pH 7.4. The circular discs containing the endothelial cells were then pinned in a specially designed chamber. Before experiments, cells were maintained in 0Ca^2+^-PSS for 2 min. 0Ca^2+^-PSS contained in mM: 140 NaCl, 5 KCl, 1 MgCl_2_, 10 glucose, and 5 Hepes at pH 7.4. All agents were applied directly to the bath along the side of the chamber, followed by pipetting gently up and down for a few times to allow quick diffusion to cells. Experiments were performed at room temperature. Fluorescence signals were recorded by a fluorescence imaging system (Olympus, Japan). If needed, the cells were pretreated with TM2E3 (10 µg/ml) or preimmune IgG (10 µg/ml) at 37°C for 2 hrs. Fura-2 fluorescence signals were recorded by a fluorescence imaging system (Olympus, Japan) with dual excitation wavelength at 340 nm and 380 nm, from which we obtained a ratio of fluorescent intensity of 340 nm to 380 nm (F 340/380). Then, [Ca^2+^]_i_ was calculated according to the ratio, using a Ca^2+^-fluorescence curve determined by Ca^2+^ standards obtained from molecular probes. The calibration solutions contains 10 ml each of 11 prediluted 10 mM K_2_EGTA/CaEGTA buffers containing 0, 1.0, 2.0, 3.0, 4.0, 5.0, 6.0, 7.0, 8.0, 9.0 and 10.0 mM CaEGTA (free Ca^2+^ ranging from 0 µM to 39 µM). All the solutions contain 100 mM KCl and 30 mM MOPS, pH 7.2.

### DNA Fragmentation Assay

The method was followed from Schwerdt’s group [41]. Cells in culture medium were collected by brief centrifugation. Then the cells were harvested in cell lysis buffer (5 mM Tris, 20 mM EDTA pH 8.0, 0.5% Triton X-100), incubated in ice for 30 min and centrifuged at 16,000 g for 20 min at 4°C. 50 µg/ml proteinase K was added into the supernatant and incubated for 60 min at 37°C. 40 µg/ml RNase A were added and incubated for 60 min at 37°C. DNA was extracted by adding the same volume phenol/chloroform/isoamylalcohol (25∶24:1). After shaking and centrifugation at 3,420 g for 30 min, the upper phase was collected. One tenth volume 3 M sodium acetate (pH 5.2) and two volumes ice cold (–20°C) ethanol were added, and the samples were turned over for several times and left overnight at –20°C. After centrifugation at 16,000 g for 20 min at 4°C, pellet was washed with 70% ice cold ethanol and dried. DNA concentration was measured at 260 nm in a spectrophotometer. DNA ladder was visualized in 2% agarose gel.

### MTT Assay

MTT (3-(4,5-dimethylthiazol-2-yl)-2,5-diphenyl tetrazolium bromide) was used to measure cell metabolic activity. Briefly, H5V cells that were stably transfected with empty vector pcDU6C, TRPM2-specific shRNA or TRPM2 were treated with indicated concentrations of H_2_O_2_ in culture media for 1 hr, or treated with TNF-α (10 ng/ml) for 36 hrs. Cells were further incubated with 250 mg MTT in phenol red free media for 2 hrs. Cells were then lysed by 10% (w/v) sodium dodecyl sulfate and purple formazan was dissolved by absolute isopropanol. The absorbance was read at OD570. Cell metabolic activity was expressed as percentage of no H_2_O_2_ (or TNF-α)-treated control. TNF-α mouse recombinant protein was purchased from Rockland, Gilbertsville, PA.

### TRPM2-specific shRNA, TRPM2 and Transfection

A 19-nt short hairpin RNA (shRNA) sequence against mouse TRPM2 gene was designed. The synthesized sequence (mouse) was: *AACCTTAGCTCATGGATTC*. The sequence was cloned into a self-constructed shRNA expression vector pcDU6C [42]. The pcDU6C contains a U6 RNA polymerase III promoter and a blasticidin resistance gene. The insertion of shRNA sequence was verified by DNA sequencing using ABI autosequencer (Perkin Elmer). Scrambled shRNA was from Santa Cruz, USA. H5V cells were transfected with empty vector pcDU6C or those containing TRPM2-specific shRNA, or the scrambled shRNA using an electroporation protocol. Under the selection pressure of 3 µg/ml blasticidin (Gibco, invitrogen, USA), the stable cell lines expressing either the TRPM2-specfic shRNA or the scrambled shRNA were established in about 10 days. Human TRPM2 cDNA (GenBank™ accession number AB001535), a generous gift from Barbara A. Miller [12], was stably overexpressed in H5V cells or HEK293 cells under Geneticin (400 µg/ml) (Gibco, invitrogen, USA) selection.

### Electrophysiology

Whole-cell membrane currents were performed at room temperature (21–25°C). HEK293 cells or H5V cells were kept in standard Ringer’s solution containing in mM: 145 NaCl, 2.8 KCl, 1 CaCl_2_, 2 MgCl_2_, 10 Glucose, 10 HEPES (pH 7.2, adjusted with NaOH). Pipette-filling solutions contained in mM: 145 Cs-glutamate, 8 NaCl, 1 MgCl_2_, 10 Cs-BAPTA, 10 HEPES (pH 7.2, adjusted with CsOH), and if appropriate, 300 µM ADP-ribose (Sigma, USA). [Ca^2+^]_i_ was buffered to 100 nM with 10 mM BAPTA and 3.6 mM CaCl_2_. Patch pipettes with a resistance of 3–5 MΩ. Pipette and membrane capacitance were automatically compensated. For H_2_O_2_ series, H_2_O_2_ (3 mM) was added into the bath solution after establishment of whole-cell configuration. Data were acquired with ‘PulseFit’ software controlling an EPC-9 amplifier (HEKA Elektronik, Lambrecht Pfalz, Germany). Cells were clamped at 0 mV. For HEK293 cells, whole-cell currents were recorded at the holding voltage of −100 mV. The currents were sampled at 25 kHz and filtered at 1 kHz. For H5V cells, whole-cell currents were recorded in linear ramp protocol from −100 mV to +100 mV with 100 ms duration immediately before H_2_O_2_ and 3–5 min after H_2_O_2_ in order for the currents to reach their peaks. Data were analyzed with PulseFit 8.7 software (HEKA).

### Western Blotting

Whole-cell lysates were extracted with protein extraction buffer, which contained in mM: 50 Tris-HCl, 150 NaCl, 50 NaF, and 1.5% Nonidet P-40, 0.5% sodium deoxycholate, pH 7.5, with addition of the protease inhibitor cocktail tablets (Roche). Protein concentrations were determined by Bradford assay. 40 µg proteins were loaded onto each lane and separated on either 8% SDS/PAGE gel after boiled in SDS loading buffer. Proteins were transferred to a PVDF membrane, and the membrane was then immersed in a blocking solution containing 5% non-fat milk and 0.1% Tween 20 in PBS for 1 hr at room temperature with constant shaking. The incubation with the primary antibody against TRPM2 (1∶3000) or TM2E3 (10 µg/ml) was carried out overnight at 4°C in PBS containing 5% non-fat milk and 0.1% Tween 20. Immunodetection was accomplished with horseradish peroxidase-conjugated secondary antibody (1∶5000), followed by ECL™ Plus Western blotting detection system (Amersham, England). Immunoblots with anti-β-tubulin antibody were used to confirm that an equal amount of proteins was loaded onto each lane. The band intensity was analyzed by ImageJ 1.42q (Wayne Rasband, USA). Active caspase-8 polyclonal antibody and active caspase-3 polyclonal antibody were purchased from Biovision, CA, USA. Pro-caspase-9 antibody was purchased from Genway Biotech, CA, USA.

### DAPI Staining

4,6-diamidino-2-phenylindole, dilactate (DAPI, dilactate) reagent (Invitrogen, USA) was used to detect the DNA condensation after H_2_O_2_ treatment. Cells were first seeded with 60–70% confluence on coverslips in DMEM supplemented with 10% Fetal Bovine Serum and a combination of penicillin-streptomycin at 37°C in a 5% CO_2_ atmosphere overnight. Cells were grown to approximately 90% confluence. 3 mM H_2_O_2_ was then added to the cells. After 24 hr, the cells were then washed with PBS for three times. Subsequently, coverslips were mounted on a microscope glass slide and stained with 1 µg/ml DAPI reagent for approximately 30 minutes in dark. The cells were then washed with PBS for three times. The morphology of cell nuclei was examined at wavelength of 340 nm under a fluorescence microscope (Olympus, Japan). The objective magnification is 20×.The exposer time is 200 ms for all groups.

### Statistics

Data are expressed as mean±standard error (S.E.). Statistical significance was determined by paired or unpaired Student’s *t*-test. For multiple comparisons, one-way ANOVA was used to determine statistical significance. *P<0.05 was considered to be statistically significant.
