# Correction: Role of TRPM2 in H_2_O_2_-Induced Cell Apoptosis in Endothelial Cells

**DOI:** 10.1371/annotation/b41e03f8-e0b8-4bf5-b36e-e0fee6364085

**Published:** 2012-10-11

**Authors:** Lei Sun, Ho-Yan Yau, Wei-Yan Wong, Ronald A. Li, Yu Huang, Xiaoqiang Yao

There was an error in the funding statement.

The correct statement is as follows: This work was supported by research grants TBRS T13-706/11, CUHK477408, CUHK479109, and CUHK478710 from Hong Kong RGC, Strategic Investment Scheme C and Group Research Grant from Chinese University of Hong Kong, and National Science Foundation of China grant 31171100. The funders had no role in study design, data collection and analysis, decision to publish, or preparation of the manuscript. 

